# Effect of Combined Perftoran and Indocyanine Green-Photodynamic Therapy on HypoxamiRs and OncomiRs in Lung Cancer Cells

**DOI:** 10.3389/fphar.2022.844104

**Published:** 2022-03-16

**Authors:** Amira M. Gamal-Eldeen, Amani A. Alrehaili, Afaf Alharthi, Bassem M. Raafat

**Affiliations:** ^1^ Clinical Laboratory Sciences Department, College of Applied Medical Sciences, Taif University, Taif, Saudi Arabia; ^2^ Radiological Sciences Department, College of Applied Medical Sciences, Taif University, Taif, Saudi Arabia

**Keywords:** Perftoran, ICG = indocyanine green, PDT—photodynamic therapy, hypoxia, hypoxamiRs, oncomirs, lung cancer

## Abstract

Indocyanine green (ICG) is a nontoxic registered photosensitizer used as a diagnostic tool and for photodynamic therapy (PDT). Hypoxia is one the main factors affecting PDT efficacy. Perfluorodecalin emulsion (Perftoran^®^) is a known oxygen carrier**.** This study investigated the effect of Perftoran^®^ on ICG/PDT efficacy in presence and absence of Perftoran^®^ *via* evaluation of phototoxicity by MTT; hypoxia estimation by pimonidazole, HIF-1*α*/*β* by ELISA, and 17 miRNAs (tumor suppressors, oncomiRs, and hypoxamiRs) were analyzed by qPCR. Compared to ICG/PDT, Perftoran^®^/ICG/PDT led to higher photocytotoxicity, inhibited pimonidazole hypoxia adducts, inhibited HIF-1*α*/*β* concentrations, induced the expression of tumor-suppressing miRNAs let-7b/d/f/g, and strongly inhibited the pro-hypoxia miRNA let-7i. Additionally, Perftoran^®^/ICG/PDT suppressed the expression of the oncomiRs miR-155, miR-30c, and miR-181a and the hypoxamiRs miR-210 and miR-21 compared to ICG/PDT. In conclusion, Perftoran^®^ induced the phototoxicity of ICG/PDT and inhibited ICG/PDT-hypoxia *via* suppressing HIF-*α*/*β*, miR-210, miR-21, let-7i, miR-15a, miR-30c, and miR-181a and by inducing the expression of let-7d/f and miR-15b.

## Introduction

The photosensitizing agent (PS), indocyanine green (ICG), is a water-soluble tricarbocyanine dye that emits fluorescence with a peak wavelength of 830 nm when illuminated with near-infrared excitation light (Schaafsma et al., 2011). ICG is a nontoxic, intravenously injectable drug and it is an FDA-approved and a registered nonspecific fluorescent probe for optical imaging diagnostic purposes such as near-infrared (NIR) image-guided oncologic surgery (Schaafsma et al., 2011). ICG has a complex molecular structure with amphiphilic properties (Houthoofd et al., 2020). Clinically, ICG is used to identify sentinel lymph node metastases in breast cancer, ophthalmic angiography, and coronary artery blood flow evaluation (Schaafsma et al., 2011). ICG is used for liver function evaluation and intraoperative local diagnosis of HCC (Ishizawa et al., 2009; Kaneko et al., 2014; Houthoofd et al., 2020). When the liver is illuminated with NIR excitation light, retained ICG emits fluorescence and, thus, HCC can be detected (Ishizawa et al., 2009). The stability of ICG accumulated in tumor tissues depends on the degree of tumor differentiation and normal tissue fibrillization around the tumor (Ishizawa et al., 2009; Kaneko et al., 2014; Kaibori et al., 2016).

Recently, ICG has been used for photodynamic therapy (PDT) which utilizes a photochemical reaction between the PS and laser light of a specific wavelength (Ishizawa et al., 2009). Briefly, PS is administered and accumulates in cancer tissues and is then irradiated with laser light directed onto the tumor. The activated PS reacts with endogenous oxygen in the surrounding tumor microenvironment (TME) resulting in the generation of reactive oxygen species (ROS), such as singlet oxygen and free radicals, which lead to cell death processes, such as apoptosis, in tumor cells. In addition, heat is generated by the reaction, and this thermal effect contributes to the tumor-suppressive effect (Oleinick et al., 2002). Similarly, when ICG is excited by NIR light, singlet oxygen can be generated. ICG is degraded by the singlet oxygen itself, and the decomposition products further decrease cell viability. These products lead to tumor cell apoptosis (Tsuda et al., 2017). Effective PDT required enough O_2_ to succeed photooxidation progression (Price et al., 2013). PDT has been primarily applied as a local therapy for malignancies such as skin cancer (Alter et al., 2015) and superficial bladder cancer (Emmanuel and Donald 2004). However, it has recently become more widely accepted as a treatment option for early gastric, esophageal, and lung cancer (Aiwu, 2016; Oinuma et al., 2016; Harris et al., 2017). Lack of O_2_ in the TME inhibits/blocks PDT; subsequently, the hypoxic TME triggers PDT-therapeutic resistance, where the aggressive hypoxic cells potentiate tumor growth, especially in solid tumors (Larue et al., 2019). Hypoxic adaptation of the TME had been recognized as an inducer of tumor expansion, invasion, and metastasis (Dang et al., 2017; Li et al., 2018) and as a key factor of therapeutic failure (Hammond et al., 2014). Improving tissue oxygenation is a growing interest in cancer therapy research studies (Larue et al., 2019), especially for PDT (Busch et al., 2000; Busch et al., 2004).

Perfluorocarbons (PFCs) are organofluorines that, due to their inert intermolecular interactions, have the capability of dissolving high volumes of gases (Larue et al., 2019). PFCs can maintain a high O_2_ concentration than the tumor matrix (Larue et al., 2019), where PFCs overcome TME hypoxia and are reported to expand PDT efficiency (Rapoport et al., 2011; Cheng et al., 2015). Liquid PFC formulas have been examined as artificial blood substitutes by exhalation for skin and lungs. Among these formulas, oxypherol (Fluosol-43) had gained FDA approval for enhancing myocardial oxygenation (Young et al., 1990), and perfluorodecalin (PFD; Perftoran^®^; Vidaphor in northern America) was clinically approved as an artificial oxygen carrier and marketed in China and Russia (Scheer et al., 2017). In Perftoran^®^, PFD is emulsified in the surfactant Proxanol 268 and electrolytes (Latson., 2019). Perftoran^®^ has been used to treat >35,000 patients of regional ischemia, hemorrhagic shock, cerebral or spinal trauma, and vascular gas embolism and indicated significant promising therapeutic results (Latson., 2019).

MicroRNAs are small non-coding RNAs (∼21–25 nucleotides in length) that interact with homologous mRNAs and regulate gene expression at the posttranscriptional stage (Bartel, 2004). Changes in miRNA expression had a major role in cancer, especially in tumor development, cell proliferation, and apoptosis. Therefore, miRNA deregulation has been involved in carcinogenesis initiation and progression, where a battery of miRNAs function as tumor-suppressor genes or oncogenes, including lung cancer biology (reviewed in Seijo et al., 2019). The therapeutic options for lung cancer, as an aggressive carcinoma, had high systemic toxicity with poor effectiveness and survival rate; accordingly, successful PDT may provide a noninvasive modality for lung cancer patients (Arbour and Riely, 2019). Therefore, the aim of this study is to explore the influence of Perftoran^®^, as an oxygen carrier, on the efficiency of ICG/PDT and on the hypoxia pathway mediators, moreover, to investigate the modulatory effect of Perftoran^®^ on hypoxia-regulating microRNAs (miRNAs), oncogenic miRNAs (oncomiRs), and tumor-suppressing miRNAs in human lung cancer cells.–Materials and Methods

Human lung adenocarcinoma epithelial A549 cells; non-small cell lung cancer (NSCLC) (ATCC, United States), were maintained in a humidified air incubator at 37°C and 5% CO_2_ and cultured in complete RPMI-1640 medium supplemented with 10% (v/v) fetal bovine serum, 1% (v/v) 100 U/ml penicillin/streptomycin, and 2 mMl-glutamine. All chemicals and reagents were obtained from (Sigma/Aldrich, VA, United States) except mentioned. Perftoran^®^ was manufactured by the Scientific Industrial Company Perftoran (Pushchino, Russia).

### Cytotoxicity

To evaluate the cytotoxicity of ICG the presence or absence of laser irradiation and Perftoran, the standard 3,4,5-dimethylthiazol-2,5-diphenyl tetrazolium bromide (MTT) method was used (Hansen et al., 1989). The cells were treated with different concentrations of ICG for 1 h, with/without a fixed concentration of Perftoran emulsion (5%), before being exposed to laser irradiation for 2 min. Perftoran^®^ was oxygenated just before treatment by bubbling of 100% oxygen through Perftoran^®^ emulsion for 5 min, using a 15 cm sterile stainless steel needle and 0.02 µm micropore filter.

In dark normoxic conditions, the laser conditions were similar to the optimum conditions that were used in our previous work (El-Daly et al., 2013). Briefly, a diode laser emitting continuous light wave (Quanta System, Milan/Italy; wavelength 807 nm, beam diameter 3.0 cm, average power 500 mW and power density 50 mW/cm^2^) was coupled with monocore optical fiber and the use of biconvex lenses to ensure adjusted power density and to ensure homogenous exposure.

### Monitoring Hypoxia

The calculated 30% of the IC_50_ value of Perftoran^®^/ICG/PDT was used to treat A549 cells, under the same dark normoxic conditions, for exploring hypoxia mediators and miRNAs. The degree of hypoxia was detected by a fluorescent pimonidazole using fluorescence microscope (Kaanders et al., 2002). Cells were mixed with Cell Lysis Solution (#LSKCLS500; Merck, United States) and protease Inhibitor Cocktail (#P8340; Merck, United States). The cell lysates were assayed by Human HIF-1*α* ELISA Fluorescent Kit (#ab229433; Abcam, Germany) and Human ARNT/HIF-1 beta Colorimetric ELISA Kit (#LS-F9594; LifeSpan Biosciences, United States) to determine the HIF-1*α* and HIF-1*β* concentrations, respectively.

### miRNA Expression

Total RNA/miRNA was extracted from cells by the miRNeasy RNA extraction kit (217004, Qiagen, Germany). Reverse transcription was performed using miScript RT II RT kit (218161, Qiagen, Germany). qRT-PCR amplification was assessed using the Stratagene Mx3000p real-time PCR system (Agilent, United States) and miScript Sybr green PCR kit (218073, Qiagen, Germany). A panel of selected miRNAs was investigated using the following Qiagen miRCURY LNA miRNA Detection probe kits: let-7a (MIMAT0010195), let-7b (MIMAT0004482), let-7c (MIMAT0004481), let-7d (MIMAT0026472), let-7e (MIMAT0004485), let-7f (MIMAT0004486), let-7g (MIMAT0004584), let-7i (MIMAT0004585), miR-15a (MIMAT0004488), miR-15b (MIMAT0004586), miR-16 (MIMAT0004518), miR-21 (MS00009079), miR-30c (MIMAT0004674), miR-34a (MIMAT0004557), miR-155 (MS00031486), miR-181a (MIMAT0000270), and miR-210 (MS00003801), and RNU6 (MS00033740). Relative miRNA expression levels were calculated using ΔΔCt method (Livak and Schmittgen, 2001), and the values were normalized to the U6 expression in non-treated controls.

### Statistical Analysis

Data were parametric and were expressed as mean ± SE. The group results were statistically analyzed by one-way ANOVA followed by a Tukey’s Post Hoc comparisons at 99% confidence interval. Significance at *p* < 0.05 was considered.

## Results

### Cytotoxicity

**FIGURE 1:**
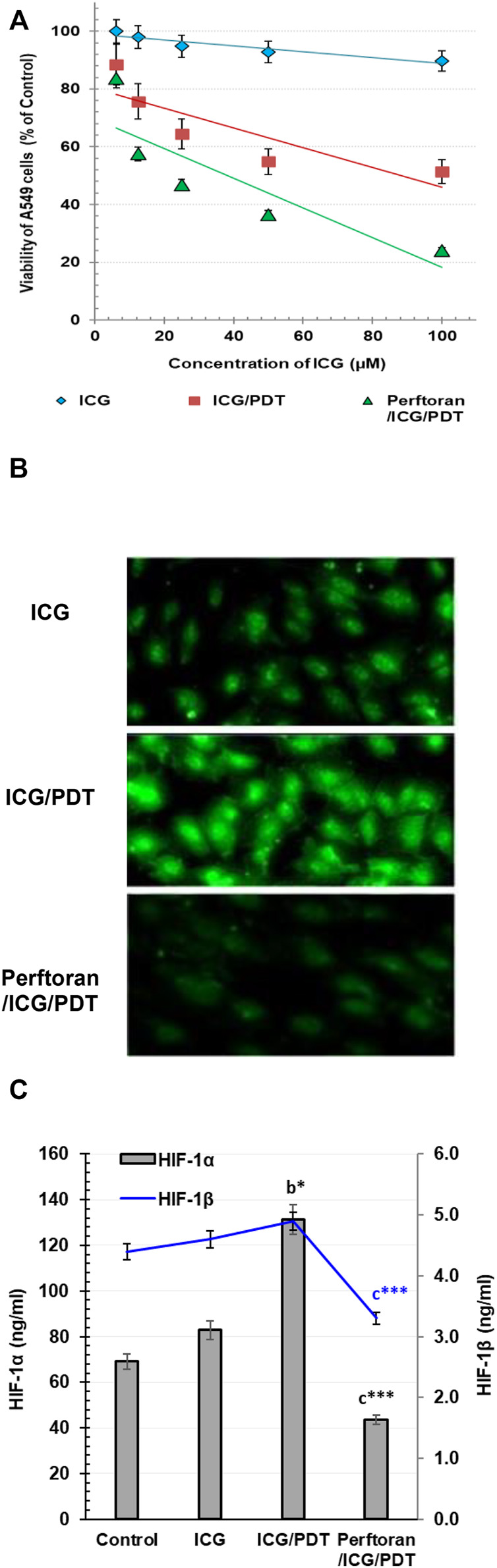
**(A)** Cytotoxicity: The viability of A549 as estimated by MTT assay. The viability is presented as % of the control (mean ± SD; *n* = 8). **(B)** Alteration in total hypoxia level in A549 cells: Detection of the change in the total hypoxia was explored by pimonidazole, which interacts with thiol groups in proteins in hypoxic cells to form green fluorescent adducts. Cells were analyzed under a fluorescence microscope (Magnification ×200). **(C)**. Determination of HIF-1*α* and HIF-1*β* proteins in A549 cells: The proteins were measured by ELISA in control cells and in ICG/PDT- and Perftoran^®^/ICG/PDT -treated cells (ng/ml; *n* = 6; mean ± SD). The results were compared to those of ^a^ control cells, ^b^ ICG-treated cells, and ^c^ ICG/PDT-treated cells; *p < 0.05, and ***p < 0.001.

Investigating the cytotoxic effect of gradual concentrations of ICG on A549 cells after 4 h revealed no cytotoxicity effect (Figure 1A); similarly, Perftoran^®^ concentration (5%) with and without laser exposure showed no cytotoxic effect in A549 cells. On the other hand, the direct photocytotoxic effect of ICG/PDT showed a corresponding decrease in cell viability leading to an IC_50_ value of 88.28 μM. In addition, the pre-incubation with Perftoran^®^ resulted in the highest photocytotoxicity with a lower IC_50_ value of 37.21 μM (Figure 1A). The calculated 30% of the IC_50_ value of Perftoran^®^/ICG/PDT was 11.16 μM, and it was used for all of the further experiments, under the same irradiation/dark/normoxic conditions and a fixed Perftoran^®^ concentration (5%).

### Monitoring Hypoxia

For the visualization of cellular hypoxia degree in A549 cells and the influence of Perftoran^®^ on this degree, confocal microscopy was utilized to analyze the fluorescent intensity after pimonidazole staining to investigate the fact that exogenous 2-nitroimidazole pimonidazole regularly interacts with thiol groups and forms fluorescent adducts in hypoxic cells (Challapalli et al., 2017). As shown in Figure 1B, the formed fluorescent adducts were lower in ICG-treated cells that were ICG/PDT-treated, which may be due to the consumed oxygen during PDT. Furthermore, the combination of ICG/PDT with Perftoran^®^ resulted in a far lower intense fluorescence in the cells, which indicated fewer hypoxia adducts. Perftoran^®^ with laser exposure showed no changes in hypoxia adducts in hypoxic cells compared with Perftoran^®^-treated hypoxic cells; data are not mentioned. In hypoxia status, HIF-1*α* is greatly involved in lowering apoptosis and increasing cell growth, while the HIF-1*β* subunit supported the consequences of HIF-1*α* activation (Ercin et al., 2019). Accordingly, to ascertain the hypoxia status, we studied the alterations in HIF-1*α* and HIF-1*β* concentrations that may interpret the inhibition of the hypoxia adducts. In this study, as shown in Figure 1C, HIF-1*α* concentration was remarkably elevated in ICG/PDT-treated cells (*p* < 0.01), which indicates a high hypoxia status that may be due the elevated consumption of O_2_ during PDT, although there was no change in HIF-1*β*. Figure 1C shows the influence of the co-treatment of ICG/PDT with Perftoran^®^ on the hypoxia mediators and indicated that Perftoran^®^, as a source of oxygenation, leads to a dramatic inhibition in HIF-1*α* and HIF-1*β* concentration (*p* < 0.001), as an indication of suppressed hypoxia. In conclusion, it can be assumed that the presence of Perftoran^®^ successfully inhibited the degree and mediators of hypoxia in A549 cells, which is suggested to be the reason for the induced phototoxicity.

### Expression of Tumor Suppressor miRNAs

**FIGURE 2:**
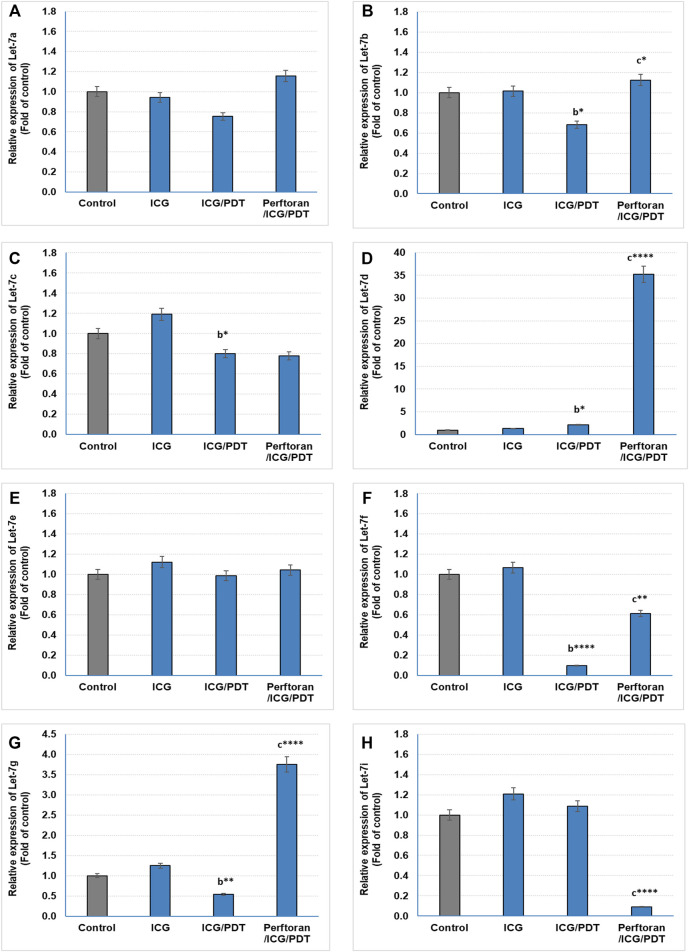
Expression of tumor suppressor miRNAs (Let-7 family; **(A–H)**): The relative expression of miRNAs was estimated by qRT-PCR. The results are expressed as fold of control. The results were compared to those of ^a^ control cells, ^b^ ICG-treated cells, and ^c^ ICG/PDT-treated cells; *p < 0.05, **p < 0.01, ***p < 0.001, and ****p < 0.0001.

**FIGURE 3:**
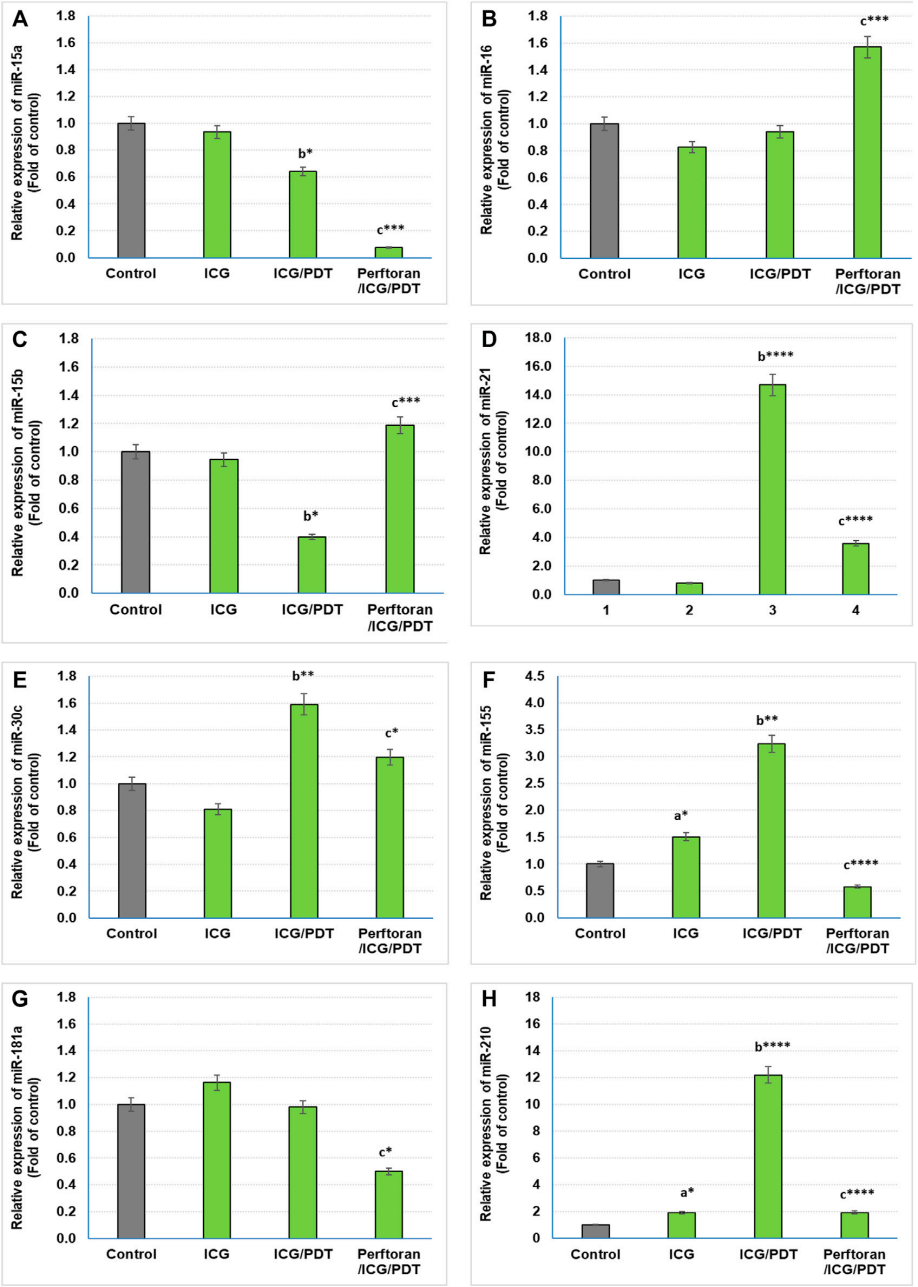
Expression of tumor suppressor miRNAs **(A,B)** and oncogenic miRNAs **(C–H)**: Relative expression of miRNAs was estimated by qRT-PCR. The results are expressed as fold of control. The results were compared to those of ^a^ control cells, ^b^ ICG-treated cells, and ^c^ ICG/PDT-treated cells; *p < 0.05, **p < 0.01, ***p < 0.001, and ****p < 0.0001.

Lethal-7 (let-7) was recognized as a regulator of cell development and proliferation. All of let-7 family (Let-7a/b/c/d/e/f/g/i and miR-98) have been identified as tumor-suppressing miRNAs that share the same 5′ ends sequence, which is essential for target recognition (Sun et al., 2014). It is reported that, in hypoxia, let-7b/e/i are upregulated and let-7a/c/d/f/g are downregulated (Nallamshetty et al., 2013). In the current study, we investigated the effect of ICG/PDT with/without Perftoran^®^ on let-7a/b/c/d/e/f/g/i expression. As shown in Figures 2A,E, neither of the cell treatments led to any changes in the relative expression of let-7a and let-7e, while ICG/PDT resulted in strong inhibition in the expression of let-7b (*p* < 0.05), let-7c (*p* < 0.05), let-7f (*p* < 0.0001), and let-7g (*p* < 0.01), Figures 2B,C,F,G. The cells that were incubated with Perftoran^®^/ICG/PDT showed high folds of expression of let-7b (*p* < 0.05), let-7d (*p* < 0.0001), let-7f (*p* < 0.01), and let-7g (*p* < 0.0001) than their corresponding members in ICG/PDT-treated cells, (Figure 2B,D,F,G). On the other hand, the expression of let-7i showed a characteristic inhibition (*p* < 0.0001) in Perftoran^®^/ICG/PDT -treated cells (Figure 2H). In addition to let-7 family, miR-15a, miR-16, and miR-34a are tumor-suppressing miRNAs. As presented in Figure 3, ICG/PDT inhibited miR-15a expression (*p* < 0.05), but not miR-16; however, unfortunately, Perftoran^®^/ICG/PDT led to dramatic inhibition in miR-15a expression. On the contrary, Perftoran^®^/ICG/PDT strongly induced the expression of miR-16 (*p* < 0.001), Figures 3A,B. Similar to let-7a and let-7e, miR-34a showed no changes after any treatment. In conclusion, the changes in the expression of let-7d/f/g/i interprets the inhibitory effect of Perftoran^®^ on hypoxia and represents its antihypoxic mechanism; moreover, the induction of the tumor suppressors let-7d, let-7g, and miR-16 shows promising findings that support the inhibition of tumor growth/development and metastasis.

### Expression of Oncogenic miRNAs

Among the known oncogenic miRNAs, this study investigated the effect of ICG/PDT with and without Perftoran^®^ on the expression of miR15b, miR-21, miR-30c, miR-155, miR-181a, and miR-210, which are in the same time either regulating hypoxia or being induced during or in response to hypoxia (Santulli, 2015; Simon, 2010; Shen et al., 2013). Our findings indicated that compared with the results of ICG/PDT-treated cells, Perftoran^®^/ICG/PDT led to a variable inhibition extent in the expression of miR-21 (*p* < *p* < 0.0001), miR-30c (*p* < 0.05), miR-181a (*p* < 0.05), miR-155 (*p* < *p* < 0.0001), and miR-210 (*p* < *p* < 0.0001), while the expression of miR-15b was unfortunately induced (*p* < 0.001), compared to ICG/PDT alone, as shown in Figures 3C–H. In conclusion, the collective inhibition of multiple oncogenic miRNAs by Perftoran^®^ may suggest that it is an inhibitor of lung tumor progress.

### Expression of HypoxamiRs

**FIGURE 4:**
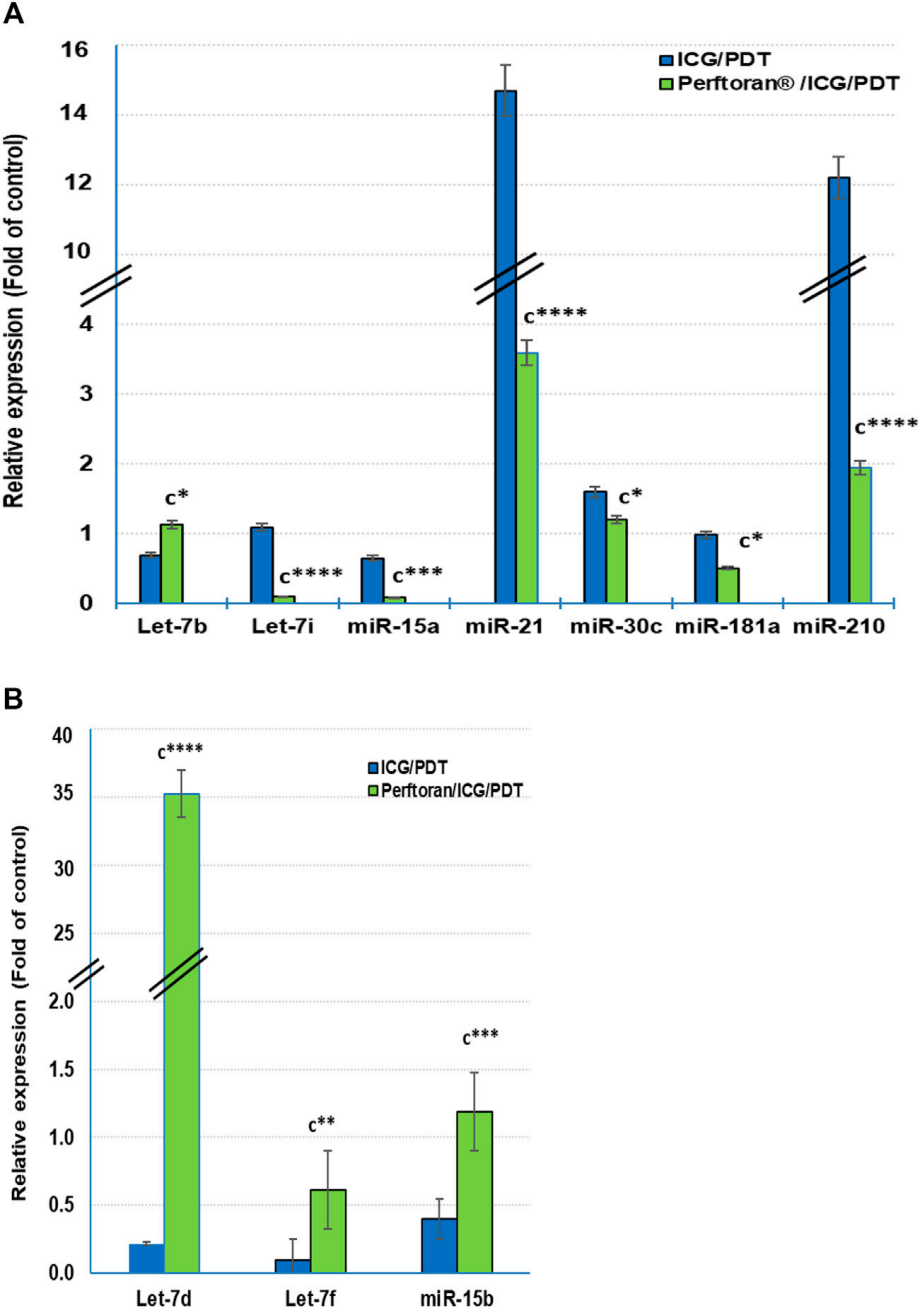
Expression of the known hypoxia-upregulated miRNAs **(A)** and the known hypoxia-downregulated miRNAs **(B)**: Relative expression of miRNAs was estimated by qRT-PCR. The results are expressed as fold of control. The results were compared to those of ^c^ ICG/PDT-treated cells; *p < 0.05, **p < 0.01, ***p < 0.001, and ****p < 0.0001.

At the cellular level, hypoxamiRs, including the master hypoxamiR miR-210, concurrently control the expression of multiple target genes that fine-tune the cellular adaptive response to hypoxia. Previously, it was documented that in hypoxia, the expressions of some miRNAs were upregulated, including, among others, let-7b/e/i, miR-15a, miR-21, miR-30c, miR-181a, and miR-210, while others were downregulated including let-7a/c/d/f and miR-15b (Simon, 2010; Shen et al., 2013; Santulli, 2015). In the current study, among the previously reported upregulated miRNAs, the presence of Perftoran^®^ with ICG/PDT successfully suppressed the hypoxia *via* the expression inhibition of let-7i (*p* < 0.0001), miR-15a (*p* < 0.01), miR-21 (*p* < *p* < 0.0001), miR-30c (*p* < 0.05), miR-181a (*p* < 0.05), and miR-210 (*p* < *p* < 0.0001) compared to ICG/PDT alone, as shown in Figure 4A. On the other hand, out of the previously reported downregulated miRNAs, the presence of Perftoran^®^ with ICG/PDT successfully induced the hypoxia *via* increasing the expression of let-7d (*p* < 0.0001), let-7f (*p* < 0.01), and miR-15b (*p* < 0.001) compared to ICG/PDT alone, as shown in Figure 4B. In conclusion, the findings suggested a strong inhibitory effect of Perftoran^®^ on hypoxia.

## Discussion

Tumor hypoxia is a consequence of the rapid growth rate of cancer cells, not allowing for appropriate angiogenesis or blood flow that diminishes oxygen and nutrients. Cancer cells, in the hypoxic spots, display a slow growth rate. The longer tumor mass remains, its resistance to anticancer drugs and metastasis ability increases (Sørensen and Horsman, 2020). In addition to the regular TME hypoxia, the consumption of the endogenous molecular oxygen by PS, during PDT, to generate ROS resulted in more hypoxia degree in the TME. This oxygen shortage in the TME stimulates multiple alterations in miRNA expression to adapt the cells to hypoxia situation, where hypoxia induces regrowth of tumor cells. Occurrence of hypoxia decreases the effectiveness of chemotherapy, radiotherapy, and PDT, which creates the demand for safe direct oxygen delivery approaches to the tumor tissues, using suitable carriers such as hemoglobin (Hb) and PFCs, a strategy to relieve hypoxic TME and improve therapeutic activity of PDT (Phung et al., 2020). Due to its extraordinary oxygen carrying capacity, PFCs have been represented as an important element in the progress of oxygen delivery approaches (Riess, 2005). In the current study, the presence of Perftoran^®^ improved the photocytotoxicity of ICG/PDT as concluded from the lower IC_50_ value (37.21 μM). The improvement may be due to the availability of the required oxygen for PDT *via* the oxygenated Perftoran^®^. The transcription factor, hypoxia inducible factor-1 (HIF-*α*), plays a dominant role in transcriptional gene regulation in hypoxia. In normoxic status, ubiquitin-dependent mechanism by oxygen-dependent prolyl hydroxylation targets the *a*-subunit and degrades HIF-1*α* (Movafagh et al., 2015). Subsequently, HIF-1*α* in its steady state with low concentration halts the transcriptional functional complex; HIF-1*α*/HIF-1*β*. On the contrary, in hypoxic status, HIF-1*α* concentration is hastily elevated and accumulated in the cytoplasm (Movafagh et al., 2015) before it arrived in the nucleus to heterodimerize with its *ß* subunit and binds to hypoxia response elements of hypoxia-regulated target genes (Pezzuto and Carico, 2018). HIF-1*α* has a regulatory function in diverse cellular pathways to control cell proliferation, apoptosis, metabolism, motility, and angiogenesis. In the present study, the improved photocytotoxicity by occurrence of Perftoran^®^ with ICG/PDT was concomitant with the strongly inhibited hypoxia pimonidazole adducts as well as the decrease in the concentration of both HIF-1*α* and HIF-1*β*.

At the transcriptional level, the hypoxic adaptations in lung vasculature are regulated by master transcription factors HIF-1*α* and HIF-2*α*, where they initiate transcription of >100 genes in hypoxia status that disturb and control the assembly of lung vascular functions; for example, angiogenesis, ROS generation/oxidative stress, proliferation, cell migration, metabolism, and survival (Chan and Loscalzo, 2010). The jumbled tumor vasculature and the formation of isolated hypoxic regions turn the development of hypoxia-relieving therapies into a real challenge (Wang et al., 2020). Therefore, our findings demonstrated that co-treatment with Perftoran^®^ led to an effective suppression of hypoxia regulators that suggested to prevent the known hypoxia-pulmonary consequences. The combination routine of HIF-1*α* inhibitors with PDT is an efficient approach for cancer therapy. Since PDT involves a high-oxygen consumption to evoke vascular destruction, this may lead to worsening of hypoxia status in the TME. Subsequently, the hypoxia-stimulated upregulation of HIF-1*α* can potentiate PDT resistance (Phung et al., 2020). The direct enrichment of oxygen in the TME, using Perftoran^®^, encouraged the inhibition of HIF-1α expression and thereafter improves the efficacy of PDT.

Several hypoxically induced miRNAs have been shown to play important roles in the hypoxic adaptation of cancer cells. The expression of let-7 was reduced in many cancers, including lung cancer, and it was linked to poor survival. The upregulation of let-7 has been proven to defeat the growth of lung cancers *in vitro*. Previous reports indicated that let-7 downregulation led to elevated oncogene RAS and MYC protein expression in lung tumors (He et al., 2010). The members of let-7 family appear to display conflicting patterns of response during hypoxia, with the caution that the findings were investigated in diverse cell types. Thus, in hypoxia, let-7g/e/i were found to be upregulated, whereas let-7a/c/d/e/f/g were suppressed, suggesting that the family members could respond to hypoxia in a cell-specific manner (Kulshreshtha et al., 2008). In the current study, ICG/PDT resulted in strong inhibition in the expression of let-7b/c/f/g. Out of these findings, the low expression of let-7 g may be responsible for the marginal induction of hypoxia adducts and HIF-*α*, compared with ICG-treated cells, after the oxygen consumption during PDT. On the other hand, Perftoran^®^/ICG/PDT led to a high expression of let-7b/d/f/g accompanied with dramatic decrease in let-7i expression, compared with that of ICG/PDT. The later diminished let-7i may be the key player in inhibiting hypoxia by Perftoran^®^/ICG/PDT, besides the highly significantly induced players (let-7d/g; *p* < 0.0001). It is known that the expression of let-7 members also influences cell-cycle regulators, such as cyclins, transcription factors, and antiapoptotic factors. In NSCLC cell lines, downregulated expression of let-7 induced cell division, whereas overexpression inhibited cell growth (Hubaux et al., 2012). This may participate in the effective cytotoxicity of Perftoran^®^/ICG/PDT, which induced the expression of many let-7 members (let-7b/d/f/g).

In addition to let-7 family, other tumor-suppressing miRNAs were investigated in the current study including: miR-15a, miR-16, and miR-34a. Among these suppressors, only miR-15a expression was inhibited by ICG/PDT, whereas adding Perftoran^®^ led to further dramatic inhibition in miR-15a expression and, on the contrary, it led to strong induction in miR-16 expression. miR-15a and miR-16 sequences and the BCL2 mRNA sequence have a complementary homology, which advocates that the Bcl2 oncoprotein is a target of posttranscriptional repression by both miRNAs (Aqeilan et al., 2010). Overexpression of the BCL2 protein has been reported in many cancers, including lung, where it acts as a key player in cancer, favoring survival by suppressing cell death (Cory and Adams, 2002). The variability in the expression of these two miRNAs, after Perftoran^®^/ICG/PDT, and their influence on BCL2 need further studies. In conclusion, the screening of tumor-suppressing miRNAs indicated that the alterations in let-7d/f/g/i expression may interpretate the suppression of hypoxia by Perftoran^®^ and suggest their role in its antihypoxic mechanism; moreover, the induction of the tumor suppressors let-7d, let-7g, and miR-16 is a promising finding that supports the suggested role of Perftoran^®^/ICG/PDT in diminishing tumor growth/development and metastasis.

The identification of miRNA oncogenes (oncomiRs) denotes raised miRNA levels in human tumor cells compared to normal cells. OncomiRs target the 3′ untranslated region (UTR), the 5′ UTR, or the coding sequence of mRNA of tumor-suppressor genes leading to their downregulation (Aqeilan et al., 2010). In the current study, the expression of the following oncomiRs was investigated: miR15b, miR-21, miR-30c, miR-155, miR-181a, and miR-210, which had been reported as either hypoxia-regulating miRNAs or induced factors during or in response to hypoxia (Simon, 2010; Shen et al., 2013; Santulli, 2015). The dramatically inhibited oncomiRs, after Perftoran^®^/ICG/PDT, were miR-21, miR-155, and miR-210 (*p* < *p* < 0.0001). It is known that miR-21 and miR-155 are among the most commonly overexpressed oncomiRs in solid tumors including lung cancer. High levels of miR-21 and miR-155 expression have been shown to predict recurrence and poor survival in NSCLC (Wang et al., 2013). As reviewed by Abd-Aziz et al., 2020 (Abd-Aziz et al., 2020), the upregulated oncomiRs (e.g., miR-21 and miR-155) increased tumorigenesis and were associated with poor prognosis and enhanced cell survival and angiogenesis. Therefore, their inhibition by Perftoran^®^/ICG/PDT is a promising finding for halting the progress of lung tumorigenesis. On the other hand, miR-15b was unfortunately induced by Perftoran^®^/ICG/PDT. miR-15b is reported to promote proliferation and invasion of non–small cell lung carcinoma cells (Wang et al., 2017), which is not noticed in the cytotoxicity results in this study. In conclusion, the suppression of multiple oncomiRs by Perftoran^®^ may suggest its promising role in the inhibition of lung tumor progress*.*

HypoxamiRs are key factors in lung cancer progression. miR-21 and miR-210, among others, are common plasma biomarkers in lung cancer patients (Shen et al., 2011). A group of three biomarker miRNAs (miR-210, miR-21, and miR-31) exhibited high sensitivity and specificity for the detection of cancerous solitary pulmonary nodules (Xing et al., 2015). Another group of six biomarker miRNAs (miR-21, miR-155, miR-210, miR-191, mir-126-5p, and miR-224) were detected in two histological phenotypes of NSCLC (Yanaihara et al., 2006). Likewise, it has been stated that the suppression of miR-21 and miR-210 led to concomitant suppression in angiogenesis and cell migration as well as invasion (Xing et al., 2015). Although miR-210 is the master hypoxamiR, multiple miRNAs were reported to be upregulated in hypoxia including, let-7b/e/i, miR-15a, miR-21, miR-30c, and miR-181a; however, other miRNAs were reported to be downregulated by let-7a/c/d/f and miR-15b, whereas the expressions of let-7e/g, miR-16, and miR-155 were varied according to the tissue type (Simon, 2010; Shen et al., 2013; Santulli, 2015). In the present study Perftoran^®^/ICG/PDT inhibited hypoxia and HIF-*α*/*β* by suppressing the expression of miR-210, miR-21, let-7i, miR-15a, miR-30c, and miR-181a, as well as inducing the expression of let-7d, let-7f, and miR-15b, compared to ICG/PDT alone.

Hypoxia is one the main factors affecting PDT efficacy. PFD, an oxygen carrier, is one of the PFC members, which in few research studies was used to enhance the efficiency of PDT *via* enrichment of the TME with oxygen, compared to other PFCs (as reviewed in Larue et al., 2019). Our study findings supported the proposed effect of PFD in enhancing the efficiency of PDT. These findings are supported by other workgroups who reported that hypericin/PDT was efficient in curing transitional cell carcinoma of the bladder (Kamuhabwa et al., 2006); PFD, was used to enhance the hypericin/PDT efficacy. They reported that absence of PFD led to poor cell death by hypericin/PDT, while its presence led to elevated oxygenation and to remarkable improvement in hypericin/PDT cell death. Moreover, the presence of PFD resulted in a higher number of apoptotic cells than hypericin/PDT alone. The report suggested that only the combined treatment leads to selective and efficient PDT. These findings are in agreement with those of the current study results. In another workgroup that dealt with ICG, Luo et al. (2019) established artificial cells by encapsulating Hb-ICG complexes into a lipid-polymer nanoparticle that permitted self-enrichment of oxygen within the cancer tissue, which further provide ROS generation during PDT, hence ensuing effective inhibition in tumor growth (Luo et al., 2016). In the last decade, several studies investigated the fitting role of some PFCs against hypoxia in PDT, as reviewed by Larue (Larue et al., 2019); however, none of them investigated PFD effect on hypoxia mediators, hypoxia-regulating miRNAs, and oncomiRs. Moreover, many reports reviewed the use of PDT in the treatment of lung cancer, including mechanisms and rationale for using variable photosensitizers and the progress in this field to cure lung cancer (Shafirstein et al., 2016). The findings of this study may provide a promising approach for a therapeutic modality to avoid hypoxia in ICG/PDT for lung cancer.

## Conclusion

Perftoran^®^ supplied the A549 cells with oxygen that supported the PDT-associated generation of ROS, which resulted in an induced photocytotoxicity of ICG/PDT and an inhibited degree of the cellular hypoxia during ICG/PDT. The inhibitory effect of Perftoran^®^ on hypoxia cascade was verified by the depletion of the hypoxia mediators HIF-*α* and HIF-*β*, and the suppression of the expression of multiple hypoxia-regulating miRNAs including; the master hypoxamiR miR-210 as well as other pro-hypoxia miRNAs (miR-21, let-7i, miR-15a, miR-30c, and miR-181a), in addition to the induction of antihypoxia miRNA expression (let-7d/f and miR-15b). Perftoran^®^ is a promising promotor for ICG/PDT effectiveness.

## Data Availability

The original contributions presented in the study are included in the article/Supplementary Material, further inquiries can be directed to the corresponding author.
